# Patient Satisfaction with the Implementation of Telehealth in Ambulatory Care during the COVID-19 Pandemic: A Single Institution Experience

**DOI:** 10.1155/2024/6800057

**Published:** 2024-02-16

**Authors:** Berhane Yohannes Hailu, Ephrem Berhe, Awol Yemane, Merhawit Atsbha Abera, Samuel Berhane, Fikaden Berhe, Saba Abraham Belay, Abraha Gebreegziabher Hailu, Bereket Berhe Abreha, Hailemariam Gebrearegay Haileeyesus, Kidu Gidey

**Affiliations:** ^1^Department of Clinical Pharmacy, School of Pharmacy, College of Health Sciences, Mekelle University, Mekelle, Ethiopia; ^2^Department of Internal Medicine, Ayder Comprehensive Specialized Hospital, Mekelle University-College of Health Sciences, Mekelle, Ethiopia; ^3^Department of Obstetrics and Gynecology, College of Health Sciences, Mekelle University, Mekelle, Ethiopia; ^4^Department of Pediatrics and Child Health, Ayder Comprehensive Specialized Hospital, College of Health Sciences, Mekelle University, Mekelle, Ethiopia; ^5^Career and Professional Development Center, College of Health Sciences, Mekelle University, Mekelle, Ethiopia

## Abstract

**Background:**

COVID-19 has reduced the capacity for delivering essential health services due to lockdown restrictions. Telehealth is an effective alternative option to improve healthcare access. However, there remain implementation challenges to patient adoption in resource-limited settings such as Ethiopia. Therefore, the purpose of this study was to assess patient satisfaction following the implementation of telehealth in ambulatory settings during the COVID-19 pandemic.

**Methods:**

A cross-sectional study was conducted at Ayder Comprehensive Specialized Hospital in the Tigray region of Northern Ethiopia. Patients who used the telehealth service were invited to participate in a patient satisfaction survey. All statistical analyses were performed using STATA Version 14.1.

**Result:**

A total of 149 patients have participated in the survey. Out of the total participants, 129 (86.6%) found that telehealth is easy to understand and overall satisfaction for telehealth was 87.9%. About two-thirds of the patients (97, 65.1%) reported that the telehealth visit is just as good as a traditional visit. The vast majority of participants (148, 98.6%) stated that they would definitely or probably use telehealth again and would recommend it to others. The majority of respondents (137, 91.9%) followed the recommendations provided.

**Conclusion:**

Patients have a high level of satisfaction with the use of telehealth during the COVID-19 pandemic. About two-thirds of patients said the telehealth visit was just as good as a traditional visit. The majority of patients followed the recommendations given to them by the healthcare providers and stated that they would definitely or probably use telehealth in the future and would recommend it to others. This high level of patient satisfaction with telehealth implementation suggests that the service could be considered in low-income countries as well.

## 1. Background

The novel coronavirus (COVID-19) has had a significant impact on healthcare service utilization and delivery practices around the world, particularly in continents with the weakest healthcare systems, such as Africa [1, 2]. Within a month of the first confirmed COVID-19 case, nearly all countries implemented rapid and stringent lockdown measures at both the national and regional levels. Such measures have helped to slow the spread of COVID-19 throughout the region [3]. However, these restrictions brought unintended consequences, as they reduced the use of essential services and healthcare visits.

According to a May 2020 World Health Organization (WHO) report, more than half (53%) of the countries surveyed have partially or completely disrupted services for hypertension treatment, 49% for diabetes and diabetes-related complications, 42% for cancer treatment, and 31% for cardiovascular emergencies [4]. Similarly, healthcare utilization in Ethiopia has declined by more than half [5, 6]. Noncommunicable disease (NCD) service disruption imposes a double burden because people living with NCDs are more likely to suffer from severe COVID-19-related illness or death. Alternative strategies for continuing care, such as telehealth, are required to combat the pandemic's impact [7]. Telehealth is described as the use of telecommunications and digital communication technologies to deliver and facilitate health and health-related services. Telehealth technologies include live video conferencing, mobile health, and remote patient monitoring [8].

During the COVID-19 pandemic, telehealth emerged as an indispensable resource for ensuring the continuity of care for patients with multiple chronic diseases [9]. While telehealth existed prior to COVID-19, the need for public health practices such as physical distancing and a desire to keep people out of high-risk settings such as hospitals accelerated the rapid shift from traditional care to telehealth [10]. Telehealth in the healthcare industry has improved patient and staff safety, care quality, pandemic management, and patient-centered care. However, its use is associated with some challenges, such as the inability to perform physical examinations, security concerns, data privacy concerns, risks to patient safety, and legal and social issues [11, 12].

Patient satisfaction is essential for the successful implementation of telehealth and for guiding action plans for telehealth service quality improvement. Telehealth has been associated with a higher level of patient and provider satisfaction [13]. Furthermore, studies have found that the quality of telehealth services and the clinical outcomes of patients following telehealth visits may be comparable to those of traditional face-to-face office visits, with the added benefit of improved access to care [14, 15]. Despite the existence of some studies on the implementation of telehealth in Ethiopia [16, 17], there has been no study on patient satisfaction with the telehealth service. Therefore, the purpose of this study was to assess patient satisfaction following the implementation of telehealth in ambulatory settings during the COVID-19 pandemic.

## 2. Materials and Methods

### 2.1. Study Setting and Period

The study was conducted at Ayder Comprehensive Specialized Hospital in the Tigray region of Northern Ethiopia. Ayder Comprehensive Specialized Hospital is one of Ethiopia's largest teaching hospitals and serves as a referral center for about 10 million people in northern Ethiopia [18]. The hospital offers a wide range of medical services to inpatients as well as outpatients. The study was conducted between April 1, 2020, and June 30, 2020.

### 2.2. Study Design and Population

A cross-sectional study was conducted to assess patients' satisfaction with the hospital's telehealth service. We implemented the telehealth service as a pilot study in April 2020, when there was a high number of COVID-19 reports, and continued it for three months. The telehealth service provided in the current study includes (1) providing education and advice for self-management, (2) linking patients to nearby health centers and pharmacies, (3) monitoring a chronic condition to detect early signs of deterioration and prompt treatment and advice, and (4) if possible, sending medications to patients.

Once the telehealth setup at Ayder Comprehensive Specialized Hospital was completed, patients were asked to call and consult with the hospital's telehealth providers. Phone numbers were announced in local media, including television, radio, and social media. In addition, phone numbers were obtained from the patients' medical records, and healthcare providers contacted the patients to assess their status. Approximately 200 patients received telehealth services over a three-month period. All the patients who received telehealth services were asked to participate in a telehealth service satisfaction survey at the end of three months, and 149 patients responded.

### 2.3. Outcome Measures

The study's main outcome of interest was the participants' level of satisfaction with the newly implemented telehealth service. Our second outcome was the patient's preference for the telehealth visit over the traditional visit.

### 2.4. Data Collection Tool

The data collection tool is a questionnaire adapted from a previous study on patient satisfaction with telehealth services [19]. The questionnaire was first translated into Tigrigna and then back into English. Although we used a validated questionnaire in other settings, it was validated in our study to adapt to the local context. An expert team evaluated the questionnaire's content validity. Two internal medicine experts and one clinical pharmacist reviewed and rated the questionnaire. The rating result was determined for each item using an item-objective congruence (IOC) score. Each question received a score of -1 (obviously not measurable), 0 (content unclear), or 1 (clearly to measure). The average IOC score of greater than 0.5 indicates strong content validity [20] and was maintained as a component of the questionnaire. The validated questionnaire was administered to 12 chronic disease patients to assess its internal consistency. The satisfaction questionnaire has a Cronbach's alpha of 0.85, indicating that the items were acceptable. The criterion for accepting Cronbach's alpha in this study was a score greater than 0.7.

Patients were interviewed over the phone to complete the patient satisfaction survey. The questionnaire included demographic information about the participants, questions about their satisfaction with telehealth, telehealth preferences, and acceptance of recommendations.

Patients rated their satisfaction with six different features using a five-point Likert scale ranging from 1 = very dissatisfied to 5 = very satisfied: overall understanding of telehealth, ease of hearing the remote practitioner, quality of the care they received, utility of the treatment and educational materials they received, convenience of the visit, and overall satisfaction. Furthermore, patients rated their telehealth visit in comparison to a traditional face-to-face medical visit as follows: better than a traditional visit, just as good as, worse, or not sure. Finally, patients used a four-point Likert scale to indicate how likely they would be to use mobile health at Ayder Hospital again and how likely they would be to recommend telehealth at Ayder Hospital to someone else.

### 2.5. Statistical Analysis

Data were entered into Epi data management (version 4.2.0) and exported to STATA version 14.1. for statistical analysis. Descriptive analysis was used to summarize the data in terms of frequency and percentages.

## 3. Results

### 3.1. Characteristics of Participants

A total of 149 patients were participated in the current study. Of the 149 participants, 21.5% were between the ages of 18 and 24, 36.9% were between the ages of 25 and 34, and only 5.4% were over 60. Approximately half of the participants had diabetes mellitus. All patients at Ayder Comprehensive Specialized Hospital used telehealth for the first time (Table 1).

### 3.2. Level of Satisfaction of the Patients

Overall, patients were very satisfied with the use of telehealth during the COVID-19 pandemic, with 86.6-90.6% reporting that they were very satisfied with all attributes. When asked about their satisfaction with the quality of care they received, 87.2% of all patients reported being very satisfied, and 89.3% said they were very satisfied with the treatment plan and educational materials they received (Table 2).

### 3.3. Preference of Telehealth and Implementation of the Recommendations

About two-thirds of patients (65.1%) said the telehealth visit was just as good as a traditional visit. Furthermore, a small percentage of patients (7.4%) indicated a preference for receiving care via telehealth. The vast majority of patients (98.6%) stated that they would definitely or probably use telehealth again and would recommend it to others. Among the 149 study participants, 91.9% followed the recommendations given to them, while the remaining 8.1% did not for different reasons (Table 3).

## 4. Discussion

Telehealth enables health professionals to deliver health services remotely by using technologies like mobile, tablets, and other similar devices. Telehealth in the healthcare industry has improved patient and staff safety, care quality, pandemic management, and patient-centered care. During the COVID-19 pandemic, we implemented telehealth services as an alternative method of healthcare delivery to improve access to healthcare. Due to the COVID-19 pandemic, the follow-up of patients with chronic diseases at Ayder Comprehensive Specialized Hospital was partially disrupted at the time we implemented telehealth. Patient satisfaction was determined as a primary outcome in this study. To the best of our knowledge, this is the first study to look into patient satisfaction with telehealth in Ethiopia. Patients were generally satisfied with the use of telehealth: 86.6-90.6% of respondents said they were very satisfied with all aspects. This is consistent with previous studies that found high levels of patient satisfaction in all components of the satisfaction survey [13, 21–23].

Almost all patients in the telehealth pilot program were very satisfied with the quality of care they received. This finding is consistent with previous studies that found telehealth visit care quality to be comparable or superior to traditional ambulatory visits, emergency department visits, or hospitalization [13, 15, 24]. In the current study, about 86.6% of the patients reported that they were very satisfied with the overall understanding of the telehealth service provided. In a similar study, 90% of patients understood their physicians' recommendations over the phone, which is comparable to our study [25].

About two-thirds of the patients (65.1%) said that a telehealth visit was just as good as a traditional visit. Consistent with our study, a study by Polinski et al. [19] found that 57% of the patients rated a telehealth visit as just as good as a traditional visit. According to the findings of a study conducted by Haxhihamza et al., the majority of respondents believe that a virtual consultation can adequately replace an in-person visit [26]. In contrast, according to Hentati et al.'s study, 62.2% of respondents did not prefer their telemedicine encounter to an in-person visit [27]. This discrepancy could be explained by differences in settings, patient diagnosis, patient characteristics, and types of virtual consultations. Nearly all of the patients said they would definitely or probably use telehealth in the future and would recommend it to others. Similar to our study, in a study by Dhahri et al., all of the patients thought the telehealth solution met their needs, and the majority of the patients chose telehealth consultation in the future [28].

Our research has important implications. Most patients were pleased with the telehealth services that were implemented; many believe that telehealth visits are just as good as traditional visits and followed the recommendations given to them. Thus, the need to expand telehealth services beyond the pandemic should be considered. This study adds to the little evidence in developing countries about the acceptability and satisfaction of telehealth services. Telehealth services should be considered as part of emergency preparedness and response to use during and after an emergency, natural disaster, or pandemic, such as COVID-19.

The study has a number of limitations. The results of the implemented telehealth survey in terms of clinical and economic outcomes have not been reported. It is also limited to a single center and may not be applicable to other institutions. The small sample size and significant nonresponse rate (>20%) were other limitations of this study. Unavailability and unstable network, short time visits, and poor internet connection were the major challenges of the study. We recommend additional research that assesses the impact of telehealth services on patient outcomes, possibly through a randomized clinical trial.

## 5. Conclusions

Patients have a high level of satisfaction with the use of telehealth during the COVID-19 pandemic. They were very satisfied with the telehealth service's understanding, ease of language and hearing, quality of care, treatment plan, and education. About two-thirds of patients said that the telehealth visit was just as good as a traditional visit. The majority of patients followed the recommendations given to them and stated that they would definitely or probably use telehealth in the future and would recommend it to others.

## Figures and Tables

**Table 1:** Characteristics of patients with telehealth visits in Ayder Comprehensive Specialized Hospital between April and June 2020 (*N* = 149).

Variables	Category	Frequency, *n* (%)
Patient age	18-24	32 (21.5)
	25-34	55 (36.9)
	35-44	23 (15.4)
	45-54	17 (11.4)
	55-64	14 (9.4)
	65 and older	8 (5.4)

Sex	Female	84 (56.4)
	Male	65 (43.6)

Have insurance	No	95 (63.8)
	Yes	54 (36.2)

Types of disease	Diabetes mellitus	70 (46.9)
	Gynecology and obstetrics cases	42 (28.10)
	Hematology	20 (13.50)
	Cardiovascular disorders	12 (8.30)
	Chronic kidney disease	5 (3.10)

First time using telehealth at Ayder or any other healthcare	Yes	100 (149)

**Table 2:** Patients' satisfaction with telehealth visits in Ayder Comprehensive Specialized Hospital between April and June 2020 (*N* = 149).

Variables	Very dissatisfied	Somewhat dissatisfied	Neither satisfied nor dissatisfied	Somewhat satisfied	Very satisfied
Overall understanding of the telehealth service	2 (1.3)	1 (0.7)	2 (1.3)	15 (10.1)	129 (86.6)
Ease of the language the practitioner used	2 (1.3)	1 (0.7)	1 (0.7)	10 (6.7)	135 (90.6)
Ease of hearing the remote practitioner	2 (1.3)	1 (0.7)	2 (1.3)	9 (6.0)	135 (90.6)
The quality of healthcare you received	2 (1.3)	1 (0.7)	5 (3.4)	11 (7.4)	130 (87.2)
The treatment plan and patient education you received	2 (1.3)	2 (1.3)	4 (2.7)	8 (5.4)	133 (89.3)
Your overall level of satisfaction with your telehealth at Ayder Hospital	2 (1.3)	2 (1.3)	3 (2.0)	11 (7.4)	131 (87.9)

**Table 3:** Patients' preferences for telehealth services and application of recommendations in Ayder Comprehensive Specialized Hospital between April and June 2020 (*N* = 149).

How did your telehealth visit compare to a traditional in-person medical visit?	
Better than a traditional visit	11 (7.4)
Just as good as a traditional visit	97 (65.1)
Worse than a traditional visit	34 (22.8)
Not sure	7 (4.7)
How likely would you be to use telehealth at Ayder Hospital again?	
Definitely will	142 (95.3)
Probably will	5 (3.3)
Probably will not	1 (0.7)
Definitely will not	1 (0.7)
How likely would you be to recommend telehealth at Ayder Hospital to someone else?	
Definitely will	142 (95.3)
Probably will	5 (3.4)
Probably will not	2 (1.3)
Definitely will not	0
Have you applied the recommendation provided through telehealth?	
Yes	137 (91.9)
No	12 (8.1)
What were the reasons for not applying the recommendations? (*n* = 149)	
Drugs were not available	6 (4.03)
Information not fitting with the previous one through the traditional visit	6 (4.03)

## Data Availability

The dataset of this study is available from the corresponding author upon reasonable request.
